# Differences in risk factors for neurophysiologically confirmed carpal tunnel syndrome and illness with similar symptoms but normal median nerve function: a case–control study

**DOI:** 10.1186/1471-2474-14-240

**Published:** 2013-08-15

**Authors:** David Coggon, Georgia Ntani, E Clare Harris, Cathy Linaker, Richard Van der Star, Cyrus Cooper, Keith T Palmer

**Affiliations:** 1 MRC Lifecourse Epidemiology Unit, University of Southampton, Southampton SO16 6YD, UK; 2 Department of Clinical Neurophysiology, Wessex Neurological Centre, Southampton General Hospital, Southampton, UK

**Keywords:** Carpal tunnel syndrome, Nerve conduction, Case–control, Obesity, Vibration, Occupation, Psychosocial, Somatising tendency, Upper limb disorders

## Abstract

**Background:**

To explore whether risk factors for neurophysiologically confirmed carpal tunnel syndrome (CTS) differ from those for sensory symptoms with normal median nerve conduction, and to test the validity and practical utility of a proposed definition for impaired median nerve conduction, we carried out a case–control study of patients referred for investigation of suspected CTS.

**Methods:**

We compared 475 patients with neurophysiological abnormality (NP+ve) according to the definition, 409 patients investigated for CTS but classed as negative on neurophysiological testing (NP-ve), and 799 controls. Exposures to risk factors were ascertained by self-administered questionnaire. Odds ratios (ORs) and 95% confidence intervals (95% CIs) were estimated by logistic regression.

**Results:**

NP+ve disease was associated with obesity, use of vibratory tools, repetitive movement of the wrist or fingers, poor mental health and workplace psychosocial stressors. NP-ve illness was also related to poor mental health and occupational psychosocial stressors, but differed from NP+ve disease in showing associations also with prolonged use of computer keyboards and tendency to somatise, and no relation to obesity. In direct comparison of NP+ve and NP-ve patients (the latter being taken as the reference category), the most notable differences were for obesity (OR 2.7, 95 % CI 1.9-3.9), somatising tendency (OR 0.6, 95% CI 0.4-0.9), diabetes (OR 1.6, 95% CI 0.9-3.1) and work with vibratory tools (OR 1.4, 95% CI 0.9-2.2).

**Conclusions:**

When viewed in the context of earlier research, our findings suggest that obesity, diabetes, use of hand-held vibratory tools, and repeated forceful movements of the wrist and hand are causes of impaired median nerve function. In addition, sensory symptoms in the hand, whether from identifiable pathology or non-specific in origin, may be rendered more prominent and distressing by hand activity, low mood, tendency to somatise, and psychosocial stressors at work. These differences in associations with risk factors support the validity of our definition of impaired median nerve conduction.

## Background

In western countries, upper limb disorders are a common cause of morbidity and disability among people of working age [1-3]. In some cases, symptoms arise from underlying pathology in the arm or neck, such as nerve entrapment or localised inflammation of soft tissues. Often, however, the pathogenesis is unclear, and the illness is classed as “non-specific”.

Prevention of upper limb disorders requires the identification of modifiable causes, and to this end, diagnostic classifications have been developed with the aim of distinguishing categories of complaint which might differ importantly in their aetiology [4-6]. In a recent systematic review, we found that for disorders at a given anatomical site (shoulder, elbow or distal arm), more complex case definitions (e.g. involving physical signs, more specific symptom patterns and/or clinical investigations) yielded similar associations with occupational risk factors to simpler definitions based only on broad groups of symptoms [7]. However, in most of the studies that we reviewed, the case definitions compared were based only on patterns of symptoms and physical signs rather than diagnostic tests, and these may not have been reliable as markers for pathogenesis. Thus, it remains possible that causes differ importantly for upper limb disorders which can be attributed to specific pathology as compared to others which affect the same anatomical site but for which there is no clear pathogenesis.

To explore whether this applies in relation to carpal tunnel syndrome (CTS), we carried out a prospective case–control study of patients referred for neurophysiological investigation because of suspected CTS, in which we distinguished case groups with and without abnormal sensory nerve conduction in the median nerve. We compared associations with various known and suspected risk factors for CTS and/or distal arm symptoms, our hypothesis being that physical risk factors would be particularly important in the development of nerve compression, whereas psychosocial risk factors would have greater impact on non-specific symptoms. We defined abnormal sensory nerve conduction according to criteria that had been developed in an earlier analysis of the inter-relation of symptoms, signs and nerve conduction velocities [8]. Our analysis therefore also provided a test of the validity and utility of this definition.

## Methods

The study population comprised adults aged 20–64 years who were resident in the catchment area served by both the neurophysiology and accident and emergency services at Southampton General Hospital. Ethical approval for the study was provided by the Southampton and South West Hampshire NHS Research Ethics Committee.

All members of the study population who attended the neurophysiology service between January 2007 and September 2009 for investigation of suspected CTS were sent a letter inviting them to answer a self-administered questionnaire and then bring it with them when they visited the hospital. In a few cases where patients failed to complete part or all of the questionnaire before coming to hospital, they were asked to do so in the clinic or to return the questionnaire later by post. The service was the only provider of nerve conduction studies for almost all of a local population of some 440,000 people, referrals coming mainly from general practitioners and orthopaedic surgeons.

Nerve conduction studies were carried out according to the normal practice of the service, by a physician or clinical physiologist trained in neurophysiology, using a Nicolet machine. Among other things, measurements were made of orthodromic sensory nerve conduction (SNC) from the index, middle and little fingers to the wrist, surface recordings being made over the median and ulnar nerves proximal to the distal wrist crease. With the patient’s permission, the findings were subsequently abstracted from the clinical record.

NP+ve cases were defined as those with at least one hand in which there had been numbness, tingling or pain in the past four weeks, and in which SNC in the median nerve was abnormal. SNC in the median nerve was considered abnormal if no signal was detectable from the index finger or if the difference in SNC velocity between the little and index fingers was > 8 m/s. This cut-point was based on an earlier comparison of the distribution of nerve conduction measurements between hands with symptoms and signs most suggestive of CTS and those with no symptoms or signs [8].

Controls were selected according to a standardised algorithm from members of the study population who attended the Accident and Emergency Department at Southampton General Hospital during the same study period, and underwent radiological examination as part of their management. They were group matched to the cases by sex and age. Controls were sent the same questionnaire as the cases, and asked to return it to the study team by post.

Our original aim was to recruit a total of at least 1000 cases and 1000 controls, which was calculated to give more than 80% power to detect a relative risk of 1.8 in a direct comparison between NP+ve and NP-ve cases for a risk factor with 10% prevalence in the latter group.

### Questionnaire

Among other things, the questionnaire asked about demographic variables; history of symptoms in the hand and arm and associated disability for everyday tasks; height and weight; ethnic origin; smoking habits; previous diagnosis of diabetes, rheumatoid arthritis or other arthritis; mental health; somatising tendency; and current or most recent occupation.

The information on height and weight was used to derive body mass index (BMI) in kg/m^2^. Mental health was evaluated through the relevant domain from the Short Form-36 (SF-36) questionnaire [9], and scores were classified to approximate thirds of the distribution in the full study sample (denoted good, intermediate and poor). Somatising tendency was assessed using elements of the Brief Symptom Inventory (BSI) [10], and was graded according to the number of common somatic symptoms from a total of five (faintness or dizziness, pains in the heart or chest, nausea or upset stomach, trouble getting breath, and hot or cold spells), that had been at least moderately distressing in the past week. The questions about current or most recent occupation covered start and finish date; whether an average working day involved each of eight specified physical activities; and various psychosocial aspects of the job including pressures from targets, bonuses or deadlines, support from a supervisor or colleagues, choice in the organisation of work, and job satisfaction.

### Statistical analysis

Statistical analysis was carried out with Stata version 11.1 software. We first excluded: subjects who fell outside the specified age range (to allow for some delay in completion of questionnaires we included subjects aged up to 65.5 years at the time of answering the questionnaire); cases who reported previous carpal tunnel surgery, who had not experienced any sensory symptoms in the hand during the past month or who had no satisfactory nerve conduction measurements in either hand; and controls with a previous diagnosis of CTS.

We then sub-divided the remaining cases according to whether or not they were neurophysiologically confirmed (NP+ve or NP-ve), and derived simple descriptive statistics for the controls and the two case groups.

Finally, we used logistic regression to explore risk factors for the two categories of case. All associations were adjusted for the group matching variables (sex and age) and for ethnic origin, and were summarised by odds ratios (ORs) and associated 95% confidence intervals (95% CIs). We first explored associations for each risk factor individually, and then used forward stepwise regression to construct a model giving mutually adjusted risk estimates for the most important risk factors. The criterion for adding a variable to the model was a p-value of < 0.2 for the reduction in deviance. Finally, we carried out similar stepwise regression to compare NP+ve cases directly with NP-ve cases, the latter being taken as the reference group.

## Results

**Figure 1:**
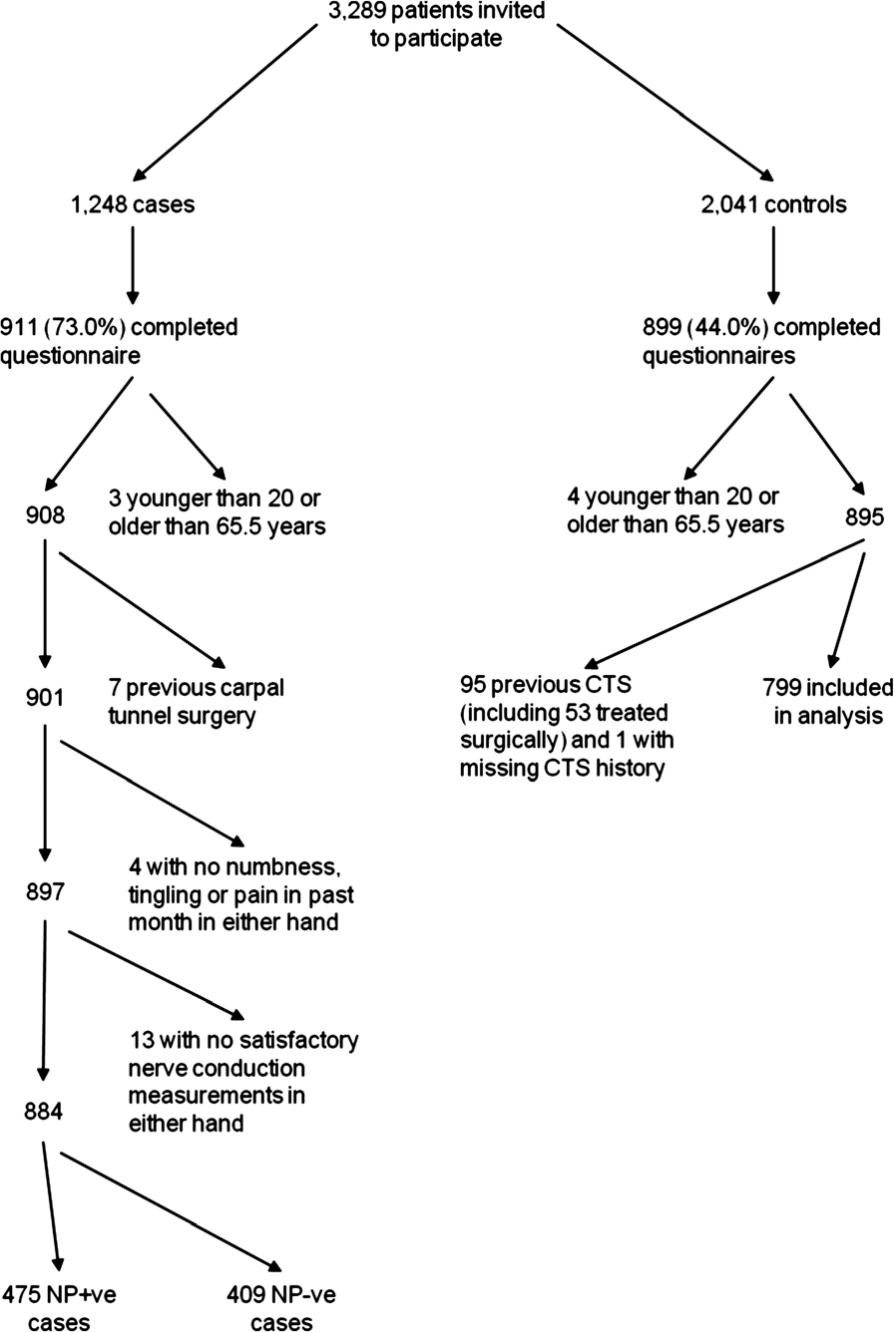
Recruitment of cases and controls and reasons for exclusion from analysis.

Over the course of the study, we approached 1248 potentially eligible cases, among whom 911 (73%) completed questionnaires (Figure 1). However, 27 were subsequently excluded because they were outside the specified age range (3), reported previous carpal tunnel surgery (7), had not experienced recent sensory symptoms in either hand (4) or had no satisfactory nerve conduction measurements in either hand (13). This left a total of 884 cases for analysis, of whom 475 were NP+ve and 409 were NP-ve.

From 2041 potentially eligible controls who were invited to take part in the study, 899 (44%) completed questionnaires, but 100 were excluded because they were outside the required age range (4), had a previous diagnosis of CTS (95 including 53 with previous carpal tunnel surgery) or missing information on history of CTS (1). Among the remaining 799 controls who were analysed, there was a broad mix of diagnoses, the most frequent categories of radiological investigation being ankle/foot (27%), wrist/hand (26%), chest (16%), other lower limb (13%) and other upper limb (12%).

Table 1 summarises the demographic characteristics of the controls and the two groups of cases. All three groups showed similar distributions by sex (predominantly female) and age (mostly 40 years or older). Most subjects were white, but there were substantially higher proportions of non-white participants among the two case groups as compared with the controls.

**Table 1:** Demographic characteristics of study participants

**Characteristics**	**Controls**		**NP+ve cases**		**NP-ve cases**	
	**n**	**(%)**	**n**	**(%)**	**n**	**(%)**
**Sex**						
Male	250	(31.3)	154	(32.4)	136	(33.3)
Female	549	(68.7)	321	(67.6)	273	(66.7)
**Age (years)**						
20-29	41	(5.1)	24	(5.1)	31	(7.6)
30-39	113	(14.1)	91	(19.2)	81	(19.8)
40-49	263	(32.9)	157	(33.1)	124	(30.3)
50-59	256	(32.0)	150	(31.6)	131	(32.0)
≥ 60	126	(15.8)	53	(11.2)	42	(10.3)
**Ethnicity**						
White	791	(99.0)	432	(90.9)	394	(96.3)
South Asian	1	(0.1)	24	(5.1)	8	(2.0)
Other	7	(0.9)	19	(4.0)	7	(1.7)

Table 2 compares hand symptoms in the two case groups. The NP+ve cases reported rather more frequent numbness, tingling or pain (74.5% v 67.7% on ≥ 14 days in the past month) and associated disturbance of sleep (44.8% v 31.3%), and somewhat longer times since last being free from hand symptoms for ≥ 4 weeks (taken as the onset of the current episode). However, the prevalence of disability for everyday tasks was similar whether neurophysiological testing was positive or negative, and recent pain at other anatomical sites in the neck and upper limb was consistently more frequent in the NP-ve cases than in the NP+ve cases.

**Table 2:** Symptoms in cases with and without neurophysiological abnormality

	**NP+ve cases**		**NP-ve cases**	
	**n**	**(%)**	**n**	**(%)**
**Days in past 4 weeks with numbness, tingling or pain in hand(s)**				
<7	21	(4.4)	28	(6.9)
7-13	49	(10.3)	53	(13.0)
14-28	354	(74.5)	277	(67.7)
Missing	51	(10.7)	51	(12.5)
**Days in past 4 weeks on which numbness, tingling or pain in hand(s) disturbed sleep**				
0	55	(11.6)	102	(24.9)
<7	89	(18.7)	77	(18.8)
7-13	83	(17.5)	78	(19.1)
14-28	213	(44.8)	128	(31.3)
Missing	35	(7.4)	24	(5.9)
**Time since completely free from numbness, tingling and pain in hands for ≥ 4 weeks**				
<6 months	161	(33.9)	164	(40.1)
≥ 6 months, <1 year	41	(8.6)	29	(7.1)
≥1 Year	254	(53.5)	196	(47.9)
Missing	19	(4.0)	20	(4.9)
**Problems in past 4 weeks because of numbness, tingling or pain in hand(s)**				
Difficulty turning taps, using kitchen gadgets, sewing or doing repairs	106	(22.4)	90	(22.0)
Having minor accidents (e.g. dropping things)	54	(11.4)	66	(16.1)
Difficulty fastening buttons or zips	57	(12.0)	54	(13.2)
Trouble writing or typing	70	(14.7)	62	(15.2)
Being very clumsy	54	(11.4)	52	(12.7)
**Pain at other anatomical sites in the past 4 weeks**				
Neck	221	(46.5)	218	(53.3)
Shoulder	214	(45.1)	217	(53.1)
Elbow	185	(39.0)	166	(40.6)

Table 3 shows associations with non-occupational risk factors, when each was analysed independently with adjustment only for sex, age and ethnicity. Risk of neurophysiologically confirmed CTS was elevated with higher BMI and worse mental health, and lower in current smokers than never or ex-smokers. In contrast, NP-ve illness showed no relation to BMI, but was significantly associated with tendency to somatise.

**Table 3:** Associations with non-occupational risk factors according to neurophysiological findings

**Risk factor**	**Controls**	**NP+ve cases**			**NP-ve cases**		
	**n**	**n**	**OR**^**a**^	**(95% CI)**	**n**	**OR**^**a**^	**(95% CI)**
**BMI (Kg/m**^**2**^**)**							
<25	303	118	1		154	1	
≥25 and <30	254	162	1.6	(1.2-2.2)	151	1.2	(0.9-1.6)
≥30	220	184	2.3	(1.7-3.1)	93	0.9	(0.7-1.2)
Missing	22	11	1.2	(0.5-2.6)	11	0.9	(0.4-1.9)
**Smoking habits**							
Never smoked	367	251	1		209	1	
Ex-smoker	210	136	1.1	(0.8-1.4)	97	0.9	(0.7-1.2)
Current smoker	213	83	0.6	(0.5-0.8)	101	0.8	(0.6-1.1)
Missing	9	5	0.6	(0.2-2.3)	2	0.4	(0.1-2.0)
**Other disease**^**b**^							
Diabetes	56	37	1.1	(0.7-1.7)	18	0.7	(0.4-1.2)
Rheumatoid arthritis	32	23	1.3	(0.8-2.3)	19	1.3	(0.7-2.3)
Other arthritis	169	92	1.0	(0.8-1.4)	92	1.3	(1.0-1.8)
**Mental health**							
Good	333	170	1		157	1	
Intermediate	236	155	1.2	(0.9-1.7)	142	1.4	(1.0-1.8)
Poor	225	148	1.3	(1.0-1.8)	108	1.1	(0.8-1.5)
Missing	5	2	0.5	(0.1-3.7)	2	0.9	(0.2-4.8)
**Number of somatic symptoms at least moderately distressing in past week**							
0	234	130	1		93	1	
1	193	123	1.1	(0.8-1.6)	110	1.5	(1.1-2.1)
≥2	372	222	1.1	(0.8-1.4)	206	1.5	(1.1-2.0)

^a^Adjusted for sex and age (in 10-year strata) and ethnicity.

^b^Risk estimates are for those with the disease relative to those who did not have it.

Table 4 gives corresponding risk estimates for occupational risk factors. The two categories of case showed significant associations of similar magnitude with prolonged, repeated movement of the wrist or fingers (other than from work with computer keyboards), repeated bending and straightening of the elbow, lack of support from supervisor or colleagues, and absence of choice in what work was done, how, and when. They were also both associated with use of vibratory tools, although the risk for NP+ve disease was higher. In addition, NP+ve disease was associated with work that entailed elevation of the hand above shoulder height, carrying weights ≥ 5 kg in one hand, and twisting of the neck for > 0.5 hours, while NP-ve illness was more common when work involved prolonged flexion of the neck.

**Table 4:** Associations with occupational risk factors according to neurophysiological findings

**Risk factor**	**Controls**	**NP+ve cases**^**a**^			**NP-ve cases**^**a**^		
	**n**^**a**^	**n**^**a**^	**OR**^**b**^	**(95% CI)**	**n**^**a**^	**OR**^**b**^	**(95% CI)**
**Physical activities in an average working day**^**c**^							
Use of keyboard or mouse for > 4 hours in total	254	120	0.7	(0.6-1.0)	145	1.1	(0.9-1.5)
Other repeated movements of wrist/fingers for > 4 hours in total	312	246	1.7	(1.3-2.1)	203	1.5	(1.2-1.9)
Repeated bending /straightening of elbow for > 1 hour in total	416	293	1.5	(1.2-1.9)	254	1.4	(1.1-1.8)
Work for >1 hour in total with tool(s) that made the hand(s) or arm(s) vibrate	60	77	2.6	(1.8-3.9)	52	1.8	(1.2-2.7)
Work with hand above shoulder height for > 1 hour in total	91	86	1.8	(1.3-2.5)	58	1.2	(0.9-1.8)
Lifting/carrying weights ≥ 5 kg in one hand	285	195	1.3	(1.0-1.6)	160	1.1	(0.9-1.5)
Work with neck bent forward for >2 hours in total	296	193	1.2	(0.9-1.5)	176	1.3	(1.0-1.7)
Work with neck twisted for > 0.5 hours in total	181	125	1.3	(1.0-1.6)	101	1.1	(0.8-1.5)
**Psychosocial risk factors**^**c**^							
Targets, bonuses or deadlines	419	247	1.0	(0.8-1.2)	207	0.9	(0.7-1.1)
Little support from supervisor or colleagues	82	78	1.7	(1.2-2.4)	78	2.0	(1.5-2.9)
Little choice in how or what work is done or in timetable and breaks	137	116	1.6	(1.2-2.1)	96	1.6	(1.2-2.1)
Job dissatisfaction	90	59	1.1	(0.8-1.5)	62	1.3	(0.9-1.9)

^a^Analyses were restricted to 1628 subjects (773 controls, 457 NP+ve cases and 398 NP-ve cases) who had held a paid job. Data on some risk factors were missing for small numbers of cases and/or controls.

^b^Adjusted for sex, age (in 10-year strata) and ethnicity.

^c^Risk estimates are for those exposed to the risk factor relative to those unexposed.

Table 5 summarises the results of the stepwise regression modelling. After adjustment for other risk factors, obesity continued to be strongly related to NP+ve disease (OR 2.1, 95% CI 1.6-2.9), as did occupational use of vibratory tools (OR 2.4, 95% CI 1.6-3.8). Positive associations were also observed with poor mental health, repeated movement of the wrist or fingers and psychosocial aspects of work, and an inverse association with smoking. For NP-ve illness, the strongest risk factors were lack of support at work (OR 1.9, 95% CI 1.3-2.7), work with vibratory tools (OR 1.6, 95% CI 1.0-2.6) and tendency to somatise (OR 1.5, 95% CI 1.1-2.1 for at least moderate distress from ≥ 2 v 0 symptoms in the past week).

**Table 5:** Associations with non-occupational and occupational risk factors in stepwise regression models

**Risk factor**	**NP+ve cases v controls**^**a**^		**NP-ve cases v controls**^**a**^		**NP+ve cases v NP-ve cases**^**a**^	
	**OR**^**b**^	**(95% CI)**	**OR**^**b**^	**(95% CI)**	**OR**^**b**^	**(95% CI)**
**BMI (Kg/m**^**2**^**)**						
<25	1				1	
≥25 and <30	1.6	(1.1-2.1)			1.3	(0.9-1.9)
≥30	2.1	(1.6-2.9)			2.7	(1.9-3.9)
**Smoking habits**						
Never smoked	1				1	
Ex-smoker	1.1	(0.8-1.4)			1.2	(0.9-1.7)
Current smoker	0.6	(0.4-0.8)			0.8	(0.5-1.1)
**Other disease**^**c**^						
Diabetes			0.7	(0.4-1.2)	1.6	(0.9-3.1)
Other arthritis					0.7	(0.5-1.0)
**Mental health**						
Good	1		1			
Intermediate	1.3	(0.9-1.7)	1.5	(1.1-2.0)		
Poor	1.4	(1.0-1.9)	1.3	(0.9-1.8)		
**Number of somatic symptoms at least moderately distressing in past week**						
0			1		1	
1			1.5	(1.1-2.2)	0.7	(0.4-1.0)
≥2			1.5	(1.1-2.1)	0.6	(0.4-0.9)
**Physical activities in an average working day**^**c**^						
Use of keyboard or mouse for > 4 hours in total			1.4	(1.1-1.9)	0.6	(0.4-0.8)
Other repeated movements of wrist/fingers for > 4 hours in total	1.5	(1.1-1.9)	1.2	(0.9-1.6)		
Repeated bending /straightening of elbow for > 1 hour in total			1.3	(0.9-1.7)		
Work for >1 hour in total with tool(s) that made the hand or arm vibrate	2.4	(1.6-3.8)	1.6	(1.0-2.6)	1.4	(0.9-2.2)
**Psychosocial risk factors**^**c**^						
Targets, bonuses or deadlines			0.8	(0.6-1.0)	1.2	(0.9-1.7)
Little support from supervisor or colleagues	1.6	(1.1-2.3)	1.9	(1.3-2.7)		
Little choice in how or what work is done or in timetable and breaks	1.4	(1.1-2.0)	1.4	(1.0-1.9)		

^a^Analyses were restricted to 1628 subjects (773 controls, 457 NP+ve cases and 398 NP-ve cases) who had held a paid job. Data on some non-occupational risk factors were missing for small numbers of cases and/or controls (see Table 3).

^b^For each of the three comparisons, risk estimates were derived from a final regression model incorporating sex, age (in 10-year strata), ethnicity, and the other variables for which results are presented.

^c^Risk estimates are for those exposed to the risk factor relative to those unexposed.

Direct comparison of the two case groups (right-hand column of Table 5) confirmed that obesity was significantly more common in the NP+ve cases (OR 2.7, 95% CI 1.9-3.9) and somatising tendency significantly less frequent (OR 0.6, 95% CI 0.4-0.9 for at least moderate distress from ≥ 2 v 0 symptoms in the past week). In addition, NP+ve disease was more strongly related to diabetes (OR 1.6) and work with vibratory tools (OR 1.4), although the differences fell just short of statistical significance.

As a check for possible bias, we re-ran the final models for Table 5, with exclusion of subjects who were not in their current or most recent job at the time when the current episode of symptoms began. Associations with occupational and other risk factors were only minimally different. For example, in the direct comparison of NP+ve with NP-ve cases, the odds ratios for obesity, prolonged occupational use of keyboards and work with vibratory tools were 2.9, 0.5 and 1.5 respectively (data not shown).

## Discussion

In this case–control study, neurophysiologically confirmed CTS showed expected associations with obesity, work with hand-held vibratory tools, and prolonged repetitive occupational movements of the wrist or fingers, as well as with poor mental health and psychosocial stressors at work. Patients with normal sensory nerve conduction reported symptoms that were broadly similar in duration and severity to those of the NP+ve cases, and like the NP+ve cases, had poorer mental health and greater exposure to occupational psychosocial stressors than controls. However, in contrast to neurophysiologically confirmed disease, illness in the absence of abnormal sensory nerve conduction was associated also with prolonged use of computer keyboards and tendency to somatise, and showed no relation to higher BMI.

The cut-point by which we defined neurophysiological abnormality was chosen to maximise discrimination between asymptomatic hands and hands with a combination of symptoms and signs most strongly suggestive of CTS [8]. However, the separation was imperfect, and abnormality by this definition was present in 25% of asymptomatic hands, while 30% of hands with strongly suggestive symptoms and signs had apparently normal sensory nerve conduction. Any misclassification of cases that resulted from this unavoidable imprecision will have tended to obscure differences between the two case groups, and not to produce spurious differential associations with risk factors.

Our control group was chosen from members of the study population who had been investigated radiologically when attending the local Accident and Emergency Department, and their response rate (44%) was short of ideal, although not atypical of what can be achieved nowadays using postal questionnaires in the UK. It is possible that the much lower prevalence of non-white patients among the controls as compared with the two case groups was an artefact of the method by which controls were selected, people from some ethnic groups being under-represented among patients attending Accident and Emergency. For this reason, all risk estimates were adjusted for ethnicity. Beyond this, however, we have no reason to suspect that our control group was importantly unrepresentative of the study population in relation to the exposures under study. Furthermore, the low response rate in the control group would not impact on the direct comparisons that were made between the two case groups. The response rate among potentially eligible cases was higher (73%), and as cases were not aware of their neurophysiological findings at the time when they agreed to take part in the study, it is unlikely that the incomplete response from cases would have caused important bias.

Although patients with a previous medical diagnosis of CTS were excluded from the control group, it is likely that the controls included some patients with sensory symptoms in the hand which had never led to medical consultation, or if they had, had not been diagnosed as CTS. To the extent that this occurred, it may have diminished differences between cases and controls, but it should not have spuriously inflated associations. Moreover, it would not have affected the direct comparisons between NP+v and NP-ve cases.

Information about exposure to risk factors was ascertained by questionnaire, and participants’ recall may not always have been accurate. If errors were non-differential (i.e. similar in cases and controls), the effect will have been to obscure associations with illness. However, it is possible that for some exposures, recall differed systematically between cases and controls. For example, the presence of hand symptoms might make people more aware of occupational activities involving use of the hands, and therefore more likely to report such activities. Again, however, such bias could not account for the differences that were observed between the two case groups.

A further limitation of the study method was the ascertainment of exposures at the time when patients presented for neurophysiological investigation, and not when their symptoms first began, which in some cases was months earlier. It is possible, for example, that some cases had changed their occupation in the interval since their illness first developed. However, when the analyses in Table 5 were restricted to the subset of cases who were in their current or most recent job when the current episode of symptoms began, associations with occupational risk factors, were virtually unchanged.

Despite the potential for errors in the ascertainment of exposures, we found clear positive associations of neurophysiologically confirmed CTS with higher BMI, occupational use of vibratory tools and repetitive work with the wrist or fingers. These findings accord with the balance of evidence in the published literature [11-22], which has led to the recognition of CTS as an occupational disease in many countries. That the odds ratio for repetitive work with the wrist and hand was only 1.5 may be a consequence of the wording of the question by which exposure was ascertained. In order to keep the questionnaire acceptably brief, we did not ask in detail about the frequency of activities, or the postures and forces entailed. The absence of an association with use of computer keyboards is also consistent with the findings overall from other research [21,23].

More controversial is the relation of CTS to smoking. In our study, risk of neurophysiologically confirmed CTS was lower in current smokers (OR 0.6, 95% CI 0.4-0.8). One earlier case–control study also found a significant inverse association with smoking [18], but in three other investigations, CTS was more common in smokers [11,13,17]. This marked heterogeneity of findings is unlikely to be attributable to chance, but might reflect unrecognised bias or confounding.

Previous research on the relation of CTS to mental health and psychosocial aspects of work has also been conflicting. A positive association with psychological distress was reported in a longitudinal study at a French footwear factory [15]; with having less influence at work in a population-based case–control study in the USA [14]; and with lack of job control and job dissatisfaction in a cross-sectional study of three occupational populations in France [24]. On the other hand, a recent systematic review found no association between any psychosocial factor and CTS [25]. Rather more consistent has been the evidence linking psychosocial stressors at work [26,27], poor mental health [28-30], and also somatising tendency [28,31-33], with upper limb musculoskeletal complaints more generally. We found that both neurophysiologically confirmed CTS and the occurrence of symptoms with normal sensory nerve conduction, were associated with poor mental health, and lack of support and choice at work. In addition, patients in the latter group exhibited stronger somatising tendency than controls. It is, of course, possible that the distress caused by sensory symptoms in the hands leads to a lowering of mood and a more negative perception of working conditions. However, some of the studies linking upper limb complaints with poor mental health and adverse psychosocial aspects of work have involved longitudinal follow-up of participants who were initially symptom-free [27-30], suggesting that reverse causation is not the full explanation for the association.

Most notable among our findings were the differential associations with obesity (significantly more common in NP+ve cases) and somatising tendency (significantly more frequent in NP-ve cases). In addition, diabetes and work with vibratory tools both tended to be more prevalent in the NP+ve cases (ORs 1.6, 95% CI 0.9-3.1, and 1.4, 95% CI 0.9-2.2, in direct comparison with NP-ve cases). There have been few attempts previously to compare risk factors in patients with suspected CTS according to neurophysiological findings, although in one large case series, BMI was a significant predictor of definite neurophysiological abnormality in patients under the age of 63 years [34]. In addition, diabetes has been linked with CTS in several studies [18,35-37], but is not widely reported as a risk factor for non-specific arm complaints. Differential associations with risk factors were also observed in a recent study from Denmark, comparing ulnar neuropathy with ulnar neuropathy-like symptoms in the absence of electrophysiological abnormality [38]. This suggested that forceful movements were a cause of injury to the ulnar nerve, but not of symptoms in the absence of such injury.

## Conclusions

The pattern of associations that we observed gives some support to our prior hypothesis that NP+ve illness would be more strongly associated with physical risk factors, and NP-ve illness with psychological risk factors. However, the difference was not completely clear-cut, and based on our findings, the hypothesis can be refined.

We propose that the physiological abnormalities associated with obesity and diabetes, and the physical stresses to tissues from use of hand-held vibratory tools and repeated forceful movements of the wrist and hand, all cause impaired function of the median nerve, which in turn can give rise to sensory symptoms in the hand. At the same time, physical activity of the hand and arm, even if insufficient to injure tissues, may aggravate sensory symptoms and/or make them more noticeable, whatever their pathogenesis. Similarly, low mood, tendency to somatise, and psychosocial stressors at work may all cause sensory symptoms, of whatever origin, to be more distressing, thereby increasing the likelihood of medical consultation and that the symptoms will be reported when those affected are questioned. However, these psychological risk factors may be relatively less important where there is a clear pathological origin of symptoms.

Further research is needed to test this theory, which could have important implications for preventive strategies. For example, if occupational activities such as prolonged use of computer keyboards act only to focus attention on symptoms, and do not injure tissues, it may be counter-productive to portray them as important health hazards requiring careful risk assessment and control, as is currently implied by legislation in the European Union [39]. Doing so may lead to unwarranted anxiety from symptoms that normally would resolve rapidly, and could thereby generate illness that would not otherwise occur. A better approach would be to present ergonomic improvements as a way of making work more pleasant and efficient, rather than as protecting against injury.

Our findings are also an encouragement to refinement of case definitions for other upper limb disorders where this can be done using criteria that are independent of symptoms and subjectively influenced physical signs such as tenderness and limitation of active movement. Moreover, they support the validity and utility of our proposed definition for abnormal median nerve conduction.

## Competing interests

The authors declare that they have no competing interests.

## Authors’ contributions

DC and KTP conceived the study and oversaw its conduct. RVdS and CC contributed to the study design. CL and ECH carried out the data collection and helped prepare the data for analysis. GN carried out the statistical analysis. DC wrote the first draft of the manuscript. All of the authors contributed to revision and finalisation of the manuscript. All authors read and approved the final manuscript.

## Pre-publication history

The pre-publication history for this paper can be accessed here:

http://www.biomedcentral.com/1471-2474/14/240/prepub
